# Supplementary material for: Thymic macrophages consist of two populations with distinct localization and origin
Source: eLife. 2022 Nov 30;11:e75148. doi: 10.7554/eLife.75148 (PMC9754631; doi:10.7554/eLife.75148)
Supplement: Transparent reporting form [file elife-75148-transrepform1.docx]

***eLife’s* transparent reporting form**

We encourage authors to provide detailed information *within their submission* to facilitate the interpretation and replication of experiments. Authors can upload supporting documentation to indicate the use of appropriate reporting guidelines for health-related research (see [EQUATOR Network](http://www.equator-network.org/%20)), life science research (see the [BioSharing Information Resource](https://biosharing.org/)), or the [ARRIVE guidelines](http://www.plosbiology.org/article/info:doi/10.1371/journal.pbio.1000412) for reporting work involving animal research. Where applicable, authors should refer to any relevant reporting standards documents in this form.

If you have any questions, please consult our Journal Policies and/or contact us: [editorial@elifesciences.org](mailto:editorial@elifesciences.org).

**Sample-size estimation**

- You should state whether an appropriate sample size was computed when the study was being designed
- You should state the statistical method of sample size computation and any required assumptions
- If no explicit power analysis was used, you should describe how you decided what sample (replicate) size (number) to use

Please outline where this information can be found within the submission (e.g., sections or figure legends), or explain why this information doesn’t apply to your submission:

No calculations were performed to predetermine sample size. Sample size was determined to be adequate based on previous experience with similar experiments and on the magnitude and consistency of measurable differences between groups in order to reproducibly detect specific effects. At least three mice were used per experimental condition. Sample size is shown in each figure legend.

**Replicates**

- You should report how often each experiment was performed
- You should include a definition of biological versus technical replication
- The data obtained should be provided and sufficient information should be provided to indicate the number of independent biological and/or technical replicates
- If you encountered any outliers, you should describe how these were handled
- Criteria for exclusion/inclusion of data should be clearly stated
- High-throughput sequence data should be uploaded before submission, with a private link for reviewers provided (these are available from both GEO and ArrayExpress)

Please outline where this information can be found within the submission (e.g., sections or figure legends), or explain why this information doesn’t apply to your submission:

In all cases N refers to independent biological replicates. One mouse without any GFP expression was excluded from the analysis in Fig. 8b. The RNA Sequencing data of thymic macrophages and thymic dendritic cells is available at NCBI Gene Expression Omnibus (GEO) as part of GSE122108 and at [www.immgen.org](http://www.immgen.org). The single cell RNA sequencing data is deposited at NCBI GEO under accession number GSE185460.

**Statistical reporting**

- Statistical analysis methods should be described and justified
- Raw data should be presented in figures whenever informative to do so (typically when N per group is less than 10)
- For each experiment, you should identify the statistical tests used, exact values of N, definitions of center, methods of multiple test correction, and dispersion and precision measures (e.g., mean, median, SD, SEM, confidence intervals; and, for the major substantive results, a measure of effect size (e.g., Pearson's r, Cohen's d)
- Report exact p-values wherever possible alongside the summary statistics and 95% confidence intervals. These should be reported for all key questions and not only when the p-value is less than 0.05.

Please outline where this information can be found within the submission (e.g., sections or figure legends), or explain why this information doesn’t apply to your submission:

Comparisons between two groups were carried out with two-tailed unpaired Student's t-test. When more than two groups were compared, a one-way ANOVA with Tukey correction was used. Differences were considered significant if p<0.05. All graphs show individual data points except for Fig. 1g and h for which N=82. Each figure and its legend explicitly state the definition of centre and error bars, the number of independent biological replicates (N), the statistical test used, and the p value.

(For large datasets, or papers with a very large number of statistical tests, you may upload a single table file with tests, Ns, etc., with reference to sections in the manuscript.)

**Group allocation**

- Indicate how samples were allocated into experimental groups (in the case of clinical studies, please specify allocation to treatment method); if randomization was used, please also state if restricted randomization was applied
- Indicate if masking was used during group allocation, data collection and/or data analysis

Please outline where this information can be found within the submission (e.g., sections or figure legends), or explain why this information doesn’t apply to your submission:

Samples were not randomized. Mice were allocated to groups based on genotype and treatment. Investigators were not blinded to mouse genotypes and treatments for planning of experiments and to ensure that appropriate sample size was achieved by sacrificing the minimum number of mice. However, blinding was used for image analysis of the in vitro phagocytosis experiments (Fig. 3C and D).

**Additional data files (“source data”)**

- We encourage you to upload relevant additional data files, such as numerical data that are represented as a graph in a figure, or as a summary table
- Where provided, these should be in the most useful format, and they can be uploaded as “Source data” files linked to a main figure or table
- Include model definition files including the full list of parameters used
- Include code used for data analysis (e.g., R, MatLab)
- Avoid stating that data files are “available upon request”

Please indicate the figures or tables for which source data files have been provided:

The source data underlying Fig. 1G and H, Fig. 3B, D, and G, Fig. 5C, F, and I, Fig. 6B, E, G, and I, Fig. 7B, C, D, and G, Fig. 8B, D, E, and F, Fig. 1S4, Fig. 2S1, Fig. 2S2, Fig. 2S3, Fig. 5S1, and Fig. 5S2 are provided in the Source Data files. All other data supporting the findings of this study are available within the article.
